# A novel classification regression method for gridded electric power consumption estimation in China

**DOI:** 10.1038/s41598-020-75543-2

**Published:** 2020-10-29

**Authors:** Mulin Chen, Hongyan Cai, Xiaohuan Yang, Cui Jin

**Affiliations:** 1 State Key Laboratory of Resources and Environmental Information System, Institute of Geographic Sciences and Natural Resources Research, Chinese Academy of Sciences, 11A, Datun Road, Chaoyang District, Beijing, 100101 China; 2 University of Chinese Academy of Sciences, Beijing, China; 3 College of Urban and Environment, Liaoning Normal University, Dalian, China

**Keywords:** Energy and society, Socioeconomic scenarios, Energy science and technology

## Abstract

Spatially explicit information on electric power consumption (EPC) is crucial for effective electricity allocation and utilization. Many studies have estimated fine-scale spatial EPC based on remotely sensed nighttime light (NTL). However, the spatial non-stationary relationship between EPC and NTL at prefectural level tends to be overlooked in existing literature. In this study, a classification regression method to estimate the gridded EPC in China based on imaging NTL via a Visible Infrared Imaging Radiometer Suite (VIIRS) was described. In addition, owing to some inherent omissions in the VIIRS NTL data, the study has employed the cubic Hermite interpolation to produce a more appropriate NTL dataset for estimation. The proposed method was compared with ordinary least squares (OLS) and geographically weighted regression (GWR) approaches. The results showed that our proposed method outperformed OLS and GWR in relative error (RE) and mean absolute percentage error (MAPE). The desirable results benefited mainly from a reasonable classification scheme that fully considered the spatial non-stationary relationship between EPC and NTL. Thus, the analysis suggested that the proposed classification regression method would enhance the accuracy of the gridded EPC estimation and provide a valuable reference predictive model for electricity consumption.

## Introduction

Electric power is essential for modern life and society. Having a clear understanding of electric power consumption (EPC) is important for infrastructure planning, energy allocation and environmental protection. However, given the commercial and confidential nature of electricity supply, detailed EPC information is difficult to obtain. Current EPC data are available mainly at administrative units, however, such data rarely meet the needs for fine scale application because of the coarse resolution. Furthermore, the data are also difficult to integrate with spatial data at other scales, and this can limit interdisciplinary applications of EPC. In contrast, gridded EPC data are more efficient in fine scales research. Therefore, developing a convenient and reliable approach for fine scale estimation of EPC is urgently needed.

Remotely sensed nighttime light (NTL) has long been considered as a direct measure of socio-economic development, and has served as primary data for a wide range of human-based research^1,2^, such as population modelling^3,4^, GDP estimation^5,6^, urban expansion mapping^7,8^, and EPC estimation^9,10^. Many studies have demonstrated a strong correlation between NTL and EPC at multiple levels, and consequently regression models for such estimation have been built^1,9,11–15^. However, the relationship between NTL and EPC varies across areas, owing to the local socioeconomic diversity^16^. In other words, the relationship between NTL and EPC is spatial non-stationary^17^, which tends to be overlooked in existing literature, where global models are often used^10,18–20^. Thus, from the spatial non-stationary perspective, local regression would be more suitable for EPC estimation. As pointed out by Shi et al.^21^, a single regression model for all administrative units is susceptible to incorrect estimation and an appropriate subdivision of administrative units could improve the estimation accuracy^22^. Some researchers have divided their study area into several sub-regions based on factors, such as geographic location^12,23^, income consideration^24^, and city function^17^. Yet, such classifications are limited in terms of accuracy, as they do not fully consider the complexity of interactions between NTL and EPC^24^. For instance, a classification based on economic factors may not sufficiently distinct the relationship between NTL and EPC, given that cities at the same level of economic development can have different energy consumption patterns and NTL levels depending on their key industries^17^. Hence, exploring an appropriate classification basis from the perspective of the relationship between EPC and NTL would provide a better understanding and a new insight for gridded EPC estimation.

In previous literature, the NTL used for EPC estimation was collected mainly from several sources including the Defense Meteorological Satellite Program Operational Linescan System (DMSP/OLS)^23,25–27^ and the day/night band (DNB) on the Visible Infrared Imaging Radiometer Suite (VIIRS) on-board the Suomi National Polar-orbiting Partnership satellite^21^. Many studies have documented that estimation accuracy would be reduced when using DMSP/OLS NTL, due to the saturation and coarse resolution^21,28,29^. In contrast, VIIRS/DNB NTL has finer resolution and better data quality than DMSP/OLS^29–32^, and consequently would provide a more reliable gridded EPC estimation^21,33^. Even so, some imperfections in the current VIIRS/DNB NTL (details given in the “Materials” section) are undesirable for gridded estimation^34^. Given these problems, more data processing steps are needed to remove the imperfections before estimation.

Against this background, the aim of this study was to develop a classification regression approach for high accuracy gridded EPC estimation. To achieve this goal, a more suitable NTL dataset for EPC estimation was first produced, based on the original VIIRS/DNB NTL. Secondly, a classification estimation scheme for the gridded EPC estimation was proposed. At the same time, apart from the proposed approach, the gridded EPC was also estimated using ordinary least squares (OLS) and geographically weighted regression (GWR). Finally, the accuracy in estimation of these three methods were compared.

## Materials

The VIIRS/DNB NTL data provided by the National Oceanic and Atmospheric Administration’s National Centers for Environmental Information (NOAA/NCEI, https://ngdc.noaa.gov/eog/download.html) were used in this study. This dataset has widely used for socioeconomic indicators spatialization owing to its fine resolution^21,31,35^. However, the data are vulnerable to stray light, resulting in emergence of distorted pixels in the mid and high latitude areas, especially in the summer^32,34,36,37^. To address this problem, two versions of the NTL are provided by NOAA, denoted by the filenames VCM (VIIRS Cloud Mask) and VCMSL (VIIRS Cloud Mask with Stray Light), respectively. The VCM version eliminates all the pixels that were contaminated by stray light, resulting in numerous missing pixels in the dataset. The VCMSL version corrects the distorted pixels through implementation of a stray light correction procedure^38^, but the data quality may be reduced in these regions^29,37,39^. Thus, both the VCM and the VCMSL data have some limitations in respect of data quality. In the present study, a more appropriate NTL dataset was produced through interpolation, which was then used for estimation purposes.

Statistical EPC data of 2015 at the prefectural level were obtained from the China City Statistical Yearbook released by the National Statistics Bureau of China. The statistical data include EPC, industrial electricity consumption (IEC) and household electricity consumption.

The land use and cover change (LUCC) data of 2015 were obtained from the land use database at a scale of 1:100,000, and collected from the Resources and Environmental Scientific Data Center (RESDC) of the Chinese Academy of Sciences (CAS). These data were interpreted by a human–computer interactive method from the Landsat TM/ ETM + images with an accuracy greater than 90%^40,41^.

## Methods

**Figure 1:**
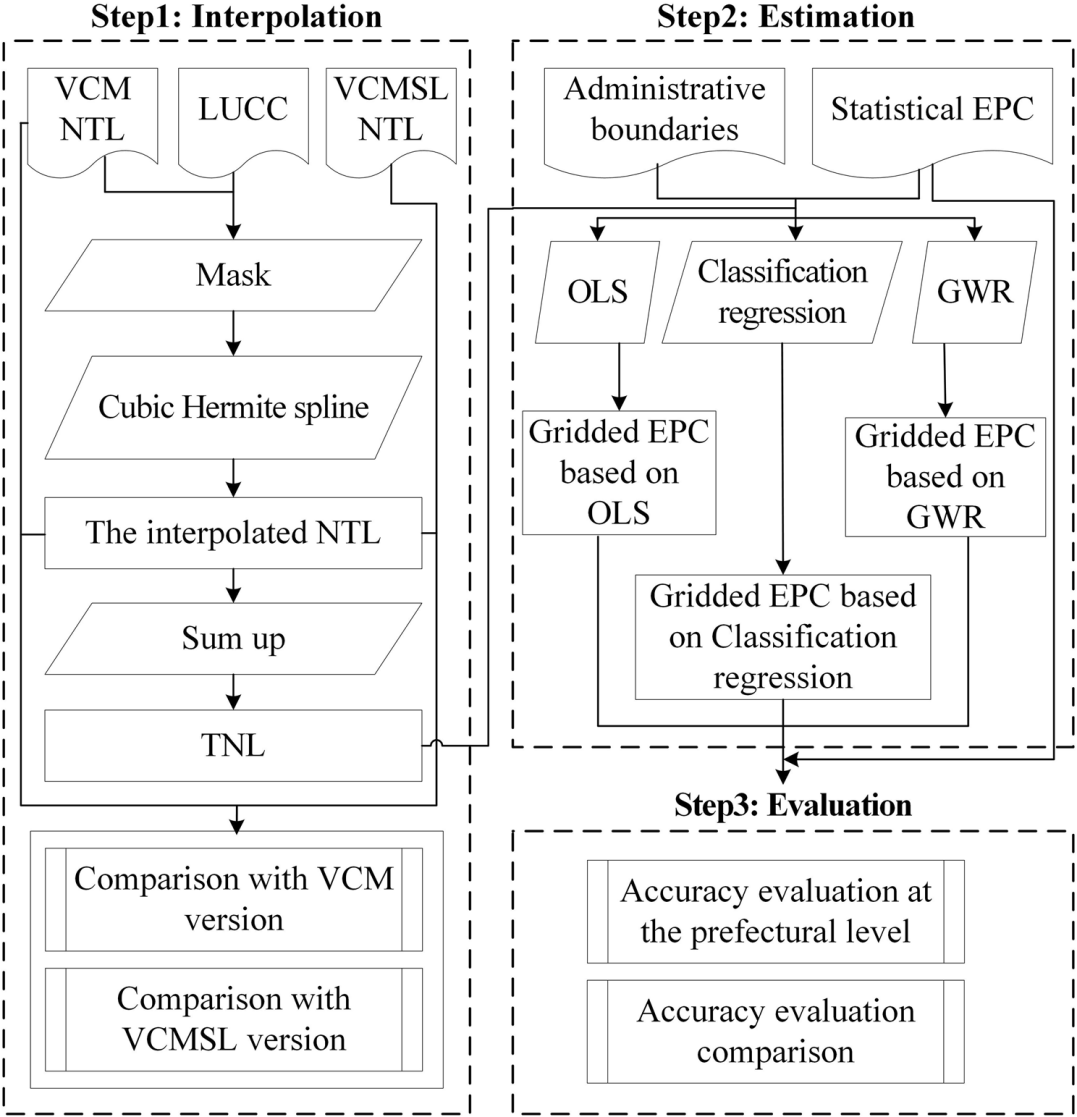
Flow chart for production and evaluation of the gridded EPC.

The methodology flow chart is displayed in Fig. 1. Given that the EPC activities occur mainly on built-up land, the EPC estimation was only performed for built-up land. First, the built-up land was extracted from the LUCC data. Second, a new NTL dataset (hereafter called the interpolated NTL) was obtained by interpolation. The annual total nighttime light (TNL) data were then composited by the interpolated NTL. Third, the gridded EPC was estimated by OLS, classification regression, and GWR, respectively. Finally, the accuracy was evaluated and compared at the prefectural level.

### Improved VIIRS/DNB NTL data production

The original monthly VIIRS/DNB NTL data are subject to stray light, and do not have background noise or ephemeral light filtered out, causing emergence of outliers such as negative pixels and abnormally high-valued pixels in the imagery^42^. Such negative effects will reduce the accuracy of estimation^21^. Therefore, an improved VIIRS/DNB NTL data, which has the outliers excluded and the missing data interpolated, was produced prior to estimation.

The threshold method was used to eliminate outliers. Theoretically, the highest level of artificial light should be emitted from the most developed areas^43^. Thus, the maximal radiation in the most developed cities in China (Beijing, Shanghai, Guangzhou and Shenzhen), which was 264.47 nW cm^−2^ sr^−1^, was determined as the threshold, and pixels with a radiation value larger than 264.47 nW cm^−2^ sr^−1^ were reassigned to that value. Meanwhile, negative-valued pixels were reassigned to zero^21^.

After filtering the outliers, the missing values were interpolated by a temporal interpolation method, as the missing pixels tend to exist over a large area. According to the study of Chen et al*.*^44^, the cubic Hermite method was used for interpolation. The monthly VCM data from August 2014 to April 2015, and from September 2015 to April 2016 were served as the initial data in interpolation proceeding at the pixel level. Subsequently, the annual TNL was calculated based on the interpolated NTL data at the pixel level (Eq. (1)), that is:

1 $$TNL_{i} = \sum\limits_{j = 1}^{12} {NTL_{ij} }$$

where *NTL*_*ij*_ is the monthly radiation value of the *i*th pixel in the *j*th month, and *TNL*_*i*_ is the annual total radiation value of the *i*th pixel.

### Regression by OLS, GWR, classification regression

Statistical EPC and TNL were regressed by OLS, GWR, and classification regression, respectively. Due to the lack of EPC statistical data in some cities, 259 cities were used for regression. OLS was given in Eq. (2).

2 $$EPC_{{\text{s}}} = a\sum {TNL_{i} + b} + e$$

where *EPC*_s_ was the statistical data of a certain city, ∑*TNL*_*i*_ was the amount of TNL for all the pixels in the corresponding areas, *a* was the regression coefficient, *b* was the intercept, and *e* was the error term. In previous studies, the intercept was assumed to be zero^10,18,24,45^. However, since NTL is not fully indicative of EPC, the intercept accounts for non-light emitting EPC. Hence, the intercept was presumed to be non-zero in this study and the *F*-test was applied to achieve statistical significance.

In consideration of the spatial spillover effect on EPC^46^, GWR was also adopted. GWR is an extension of linear regression model^47^. It uses some distance-based weighting function to permit the coefficients to vary locally within the bandwidth^48^. GWR was performed by ArcGIS 10.2 and the bandwidth was determined by AICc^49^.

**Figure 2:**
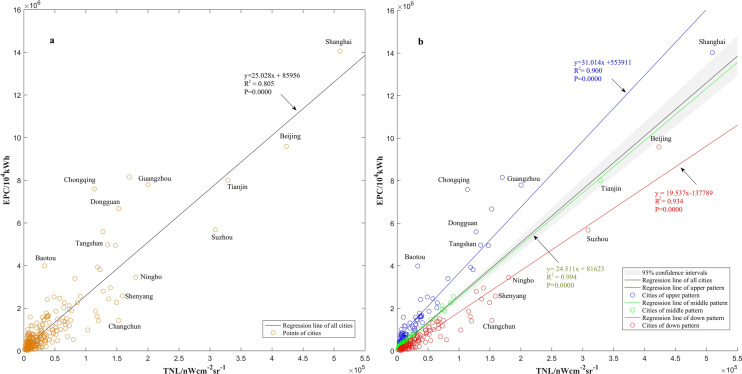
Regression of TNL and EPC by OLS and the proposed classification regression. (**a**) OLS. (**b**) Classification regression.

**Figure 3:**
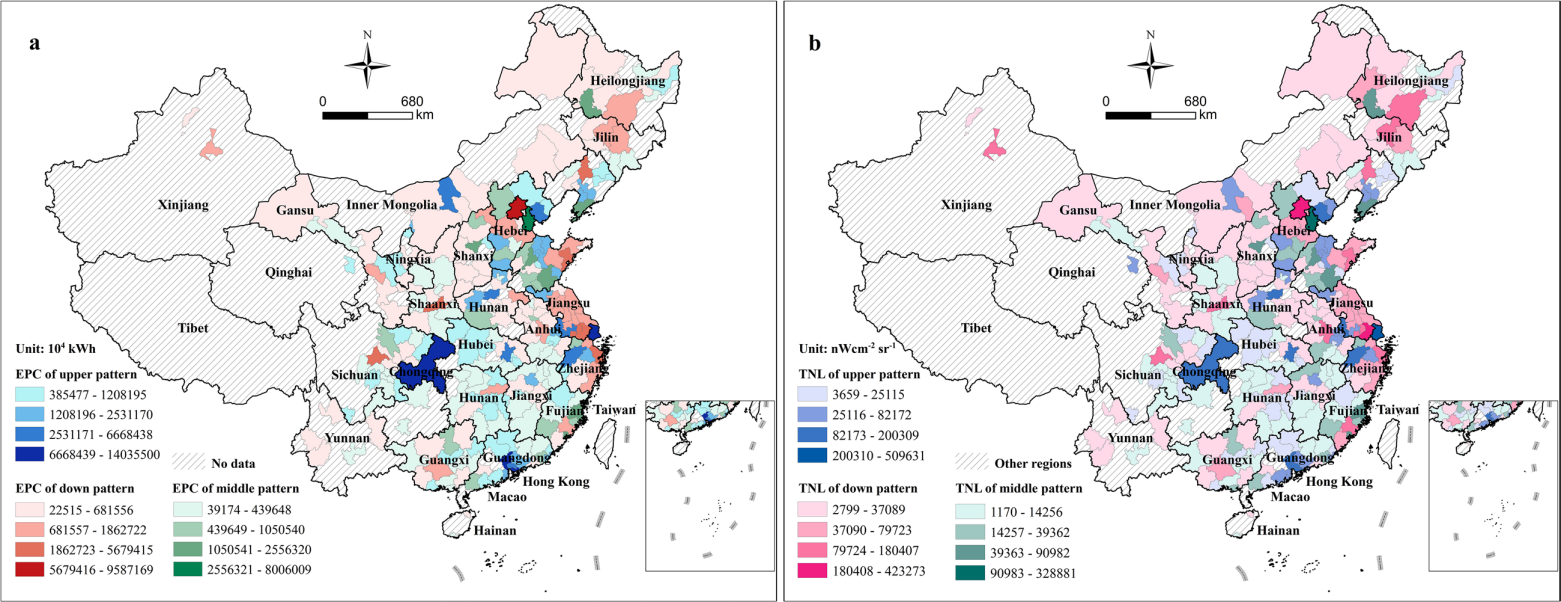
Distributions of the three observed patterns. (**a**) EPC in the three observed patterns. (**b**) TNL in the three observed patterns. The map was generated by ArcGIS 10.2 (https://www.arcgis.com/index.html).

The classification regression was built based on OLS. In order to maximize the stationary aspect of the relationship between EPC and NTL, the classification regression grouped the cities into three patterns based on the 95% confidence interval of the OLS regression coefficients, and then each pattern was regressed by Eq. (2), respectively. The three patterns were: the upper pattern, which consists of cities that were over the 95% confidence interval (72 cities, blue points in Fig. 2b); the middle pattern, which consists of cities that were within the confidence interval (78 cities, green points in Fig. 2b); and the down pattern, which consists of cities that were under the confidence interval (109 cities, red points in Fig. 2b). In general, the upper pattern displayed a low TNL with a relative high EPC, the down pattern featured a high TNL with a relative low EPC, and the middle pattern was in between these two. EPC and TNL spatial distributions of the three patterns were displayed in Fig. 3.

### Gridded EPC estimation

The gridded EPC for the three assessing methods was calculated using Eqs. (3)–(5). It was assumed that all the grids in a certain city had a segmented intercept based on their weight. Furthermore, IEC varies significantly between the industrial and non-industrial regions within a city. Thus, this difference was taken into consideration in the estimation.

3 $$EPC_{{{\text{G}}i}} = \left\{ {\begin{array}{*{20}l} {aTNL_{i} + b_{1} \frac{{TNL_{i} }}{{\sum {TNL_{i} } }}} \hfill & {\text{industrial region}} \hfill \\ {aTNL_{i} + b_{2} \frac{{TNL_{i} }}{{\sum {TNL_{i} } }}} \hfill & {\text{non } - \text{ industrial region}} \hfill \\ \end{array} } \right.$$

4 $$b_{1} = b\frac{{IEC_{{\text{s}}} }}{{EPC_{{\text{s}}} }}$$

5 $$b_{2} = b{ - }b_{1}$$

where *EPC*_G*i*_ was the estimated EPC of the *i*th grid in a certain city. The values of *a* and *b* were obtained from the three regression methods. *IEC*_s_ and *EPC*_s_ represented the statistical IEC and EPC of the city. *TNL*_*i*_ was the annual total nighttime light radiation of the *i*th grid, and *∑TNL*_*i*_ was the city’s aggregated TNL.

### Accuracy evaluation

The accuracy at the prefectural level was evaluated using relative error (RE) (Eq. (6)) and mean absolute percentage error (MAPE) (Eq. (7)).

6 $$RE = \frac{{EPC_{{\text{e}}} - EPC_{{\text{s}}} }}{{EPC_{{\text{s}}} }} \times 100\%$$

7 $$MAPE = \frac{1}{n}\sum\limits_{i = 1}^{n} {\left| {\frac{{\mathop {EPC}\nolimits_{{{\text{e}}i}} - \mathop {EPC}\nolimits_{{{\text{s}}i}} }}{{\mathop {EPC}\nolimits_{{{\text{s}}i}} }}} \right|} \times 100\%$$

where *EPC*_e_ represented the estimated EPC and *EPC*_s_ represented the statistical EPC. *i* represented the *i*th city. *n* represented the total number of cities and equaled to 259 in present study. The closer RE and MAPE are to zero, the more accurate the estimation is.

## Results

### Evaluation of the interpolated NTL data

**Figure 4:**
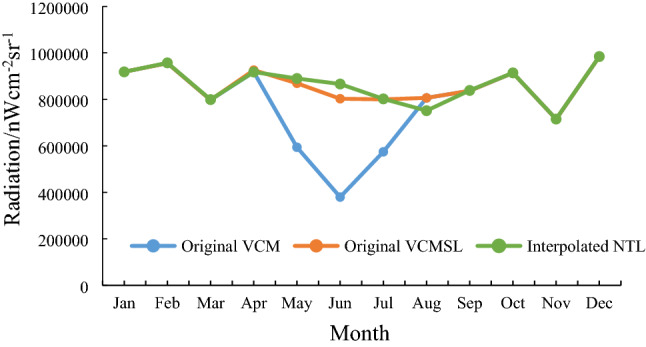
Monthly total nighttime light (TNL) time series at the national level for the original visible infrared imaging radiometer suite cloud mask (VCM) version, the original visible infrared imaging radiometer suite cloud mask with stray light (VCMSL) version and the interpolated nighttime light (NTL) data.

The monthly TNL time series for the original VCM, VCMSL and the interpolated NTL data at the national scale were compared and shown in Fig. 4. The TNL data for the VCM version in the summer months were much lower than those in other months, resulting in dramatic fluctuations in the time series curve. In contrast, the VCMSL and interpolated versions were smoother. If a certain region did not suffered from any severe events, such as natural disasters or power outages, but its TNL fluctuated dramatically, it could be inferred that its NTL data did not reflect the real socio-economic conditions^50,51^, and, therefore, were inappropriate for robust socio-economic research. From this standpoint, the interpolated NTL data are more suitable for estimation of the EPC than the VCM data.

**Figure 5:**
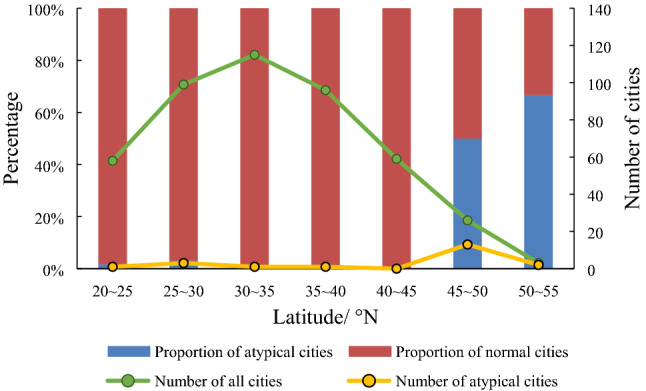
Frequency of occurrence of the sudden TNL drop for the visible infrared imaging radiometer suite cloud mask with stray light (VCMSL) product. Cities that had the sudden drop were called the atypical cities. The “number of all cities” is the total number of cities within the corresponding latitude zone.

As shown in Fig. 4, there was only a slight difference between the VCMSL version and the interpolation version at the national level. Thus, a further comparison of these two versions was performed at the prefectural level. The results demonstrated that the interpolated NTL data performed better than the VCMSL product in the mid and high latitude areas. The TNL values for some cities in the VCMSL version appeared to be susceptible to sudden sharp drops in certain months, but this feature did not show up in the interpolated NTL data. For reporting purposes, we defined cities where the TNL values suddenly became zero as being atypical cities. A latitudinal statistic was used to calculate the frequency of the atypical cities (Fig. 5). Since a high ratio of 50% came from the 45–50° N (13 cities) and 50–55° N (66.7%, 2 cities) regions, the atypical cities tended to occur in the mid-high latitude regions where stray light shows severe contamination. In comparison, the atypical cities were not evident in the interpolated NTL dataset. This difference in the respective NTL datasets most likely results from the different processes used to produce the two NTL versions. The VCMSL NTL was produced by a stray light correction procedure^52^, and, consequently, the mid-high latitude areas, where there was severe stray light contamination, most likely was susceptible to this problem^34^, whereas the missing pixels were corrected based on the long, historical, time series data in the interpolated NTL, which could effectively avoid dramatic TNL time series changes.

### Evaluation of the gridded EPC estimation

**Figure 6:**
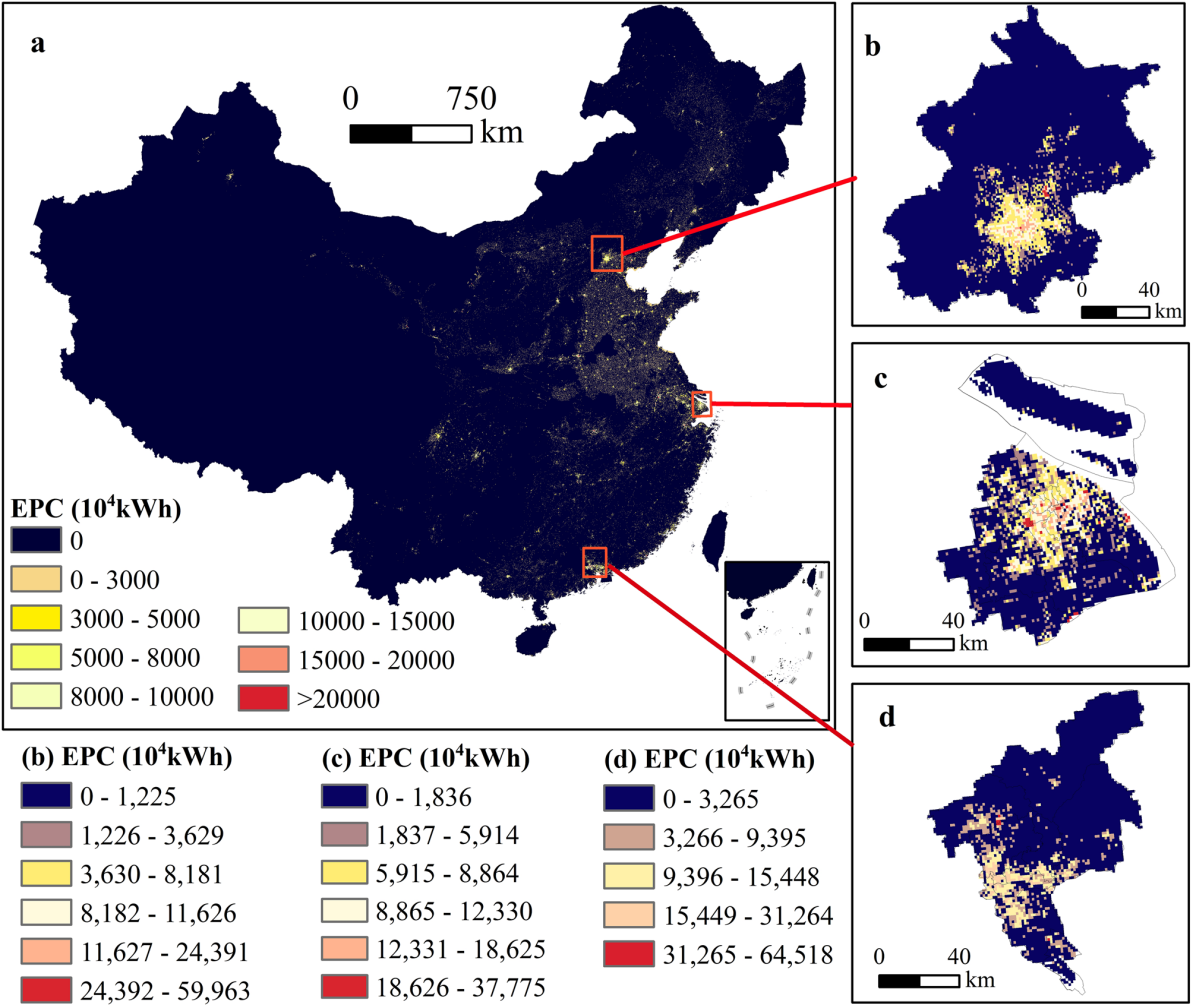
Distribution of the gridded EPC based on the classification regression method. (**a**) Spatial distribution of gridded EPC in China. Gridded EPC distribution in Beijing, Shanghai and Guangzhou is displayed in (**b**–**d**), respectively. The map was generated by ArcGIS 10.2 (https://www.arcgis.com/index.html).

The gridded EPC based on classification regression was shown in Fig. 6. The EPC was concentrated in eastern China. The high EPC areas were centered on megacities and provincial capitals, especially Shanghai, Guangzhou and Beijing. The low TNL areas were mostly distributed in rural regions and less developed satellite cities. Within a city, the highest EPC tended to occur mainly at airports, railway stations and business districts.

The accuracy of the evaluated results at the prefectural level is shown in Table 1. There were 145 cities whose RE values fell into the [− 25%, 25%] bin when adopting the classification estimation, while there were only 72 cities in that bin for OLS and 86 for GWR. Furthermore, the number of cities for which the absolute RE values exceeded 50% decreased from over a hundred to 62 after classification. The lowest MAPE values for the proposed classification estimation also demonstrated that the approach resulted in a significant improvement in accuracy compared to OLS and GWR.

**Table 1:** Number of cities in the different relative error (RE) bins and mean absolute percentage error (MAPE) values for each of the three estimation methods.

	OLS	GWR	Classification regression
|RE|≤ 25%	72	86	145
25% <|RE|≤ 50%	71	70	52
50% <|RE|	116	103	62
MAPE	57.1%	73.9%	38.0%

## Discussion

Different from most previous literature, a novel classification regression method that fully considered the spatial non-stationary relationship between the EPC and NTL was described. The results indicated that the proposed method performed best among the three assessing methods (Table 1). Compared to OLS, the relationship between the EPC and NTL was stronger for the classification regression, as revealed by the R^2^ values (0.900, 0.994 and 0.934 *vs*. 0.805). Moreover, a previous study by Shi et al.^21^ also confirmed this finding. In that study, the VIIRS/DNB NTL data were also applied to gridded EPC estimation based on OLS. The RE values in the present work were significantly reduced compared to that study, notably in Anhui, Hebei, Heilongjiang and Shaanxi provinces. In addition, GWR is powerful for spatial non-stationary parameters regression^53^. However, the Moran’s bivariate spatial autocorrelation index of statistical EPC and TNL was only 0.070. It was evident that the spatial autocorrelation between EPC and TNL was weak, in other words, the distance between cities has little influence on the regression. Thereby, the distance-based weighting scheme was not likely to render a better performance than the proposed method.

The favorable results benefited mainly from a reasonable classification scheme being realized. The classification basis is important to high accuracy estimation. In a study of Li et al.^17^, cities were grouped into technology and education cities, industrial cities, and service-oriented cities. However, the classification basis based on city function still have some limitations on ensuring the spatial stationary relationship between EPC and TNL. For example, Changchun, Shenyang, Baotou, Dongguan were the famous industrial cities in China, but differed greatly in the relationship between EPC and TNL (Fig. 2). In part because EPC used for different purposes would emit different levels of light. Industrial electricity consumed for electrolysis, heating and other energy-intensive processes, has a little light emitted^22^, whereas industrial electricity consumed for oil, natural gas, and coal mineral resources mining, has bright light emitted due to the mining lights and industrial fires^22^. It leads to different key industries playing a role in the relationship between EPC and TNL^17^. The classification basis based on city function, thus, was insufficient for spatial stationary classification of EPC and TNL; instead, the classification scheme we proposed effectively avoided ambiguous classification.

**Figure 7:**
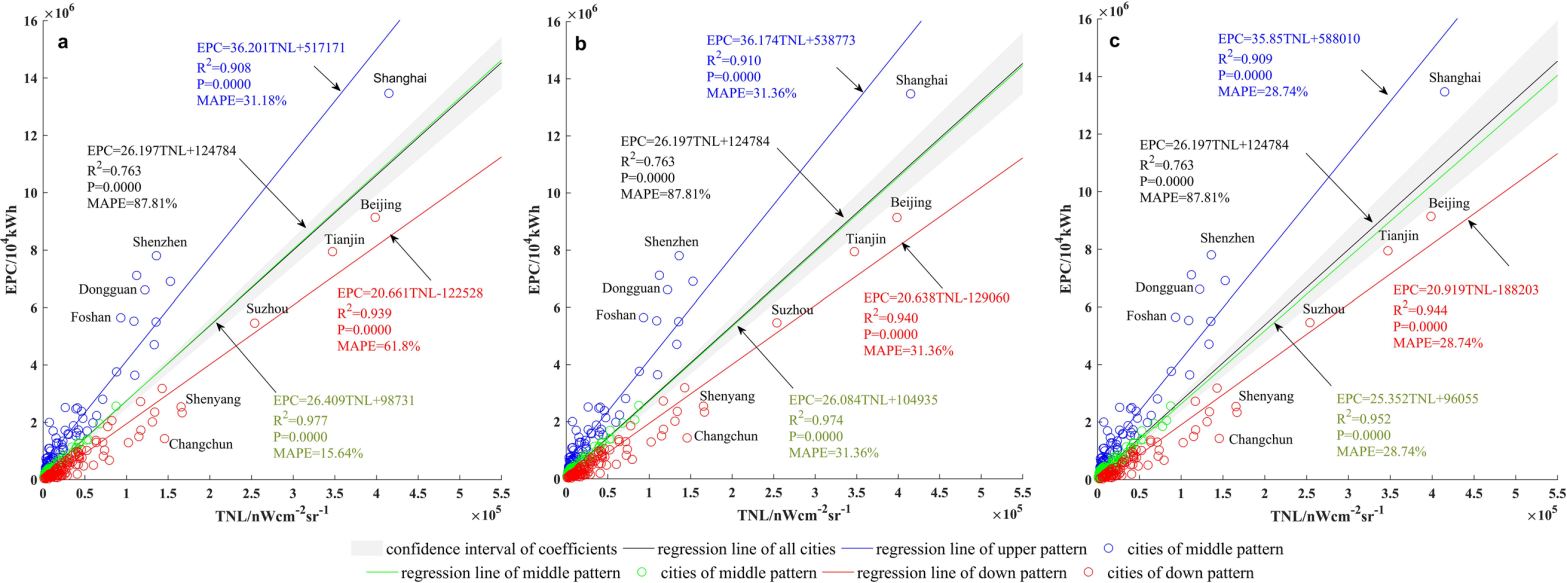
Classification regression based on different confidence intervals. (**a**) 90% confidence interval. (**b**) 95% confidence interval. (**c**) 99% confidence interval.

The threshold of confidence interval may cause uncertainty of the classification method. Thus, apart from 95% confidence interval, 90% and 99% confidence intervals were also served as the threshold, respectively. As the comparison results shown, whether the regression coefficients, or the MAPE values, the differences were slight (Fig. 7). It was because for the most cities, their patterns did not change as the confidence intervals changed, and, consequently, the classification regression method was seldom affected by the threshold of confidence interval. Thus, from this perspective, the classification regression method was robust.

Inevitably, some limitations existed when estimating the gridded EPC based on the VIIRS/DNB NTL. First, the overpass time of the SNPP satellite is after midnight and near 01:30 am^54^. The outdoor artificial light is low at this time, leading to much EPC that is not reflected in the NTL dataset. Second, monthly changes in vegetation and snow cover are also contained in the current VIIRS/DNB NTL dataset. These physical changes are known to be undesirable for socio-economic studies^55^. Despite this, given that China is mainly located in the low-mid latitude regions, most parts of the country would not be impacted by that seasonal noise^56^, and, consequently, the EPC estimation result is considered reliable. However, for high latitude areas, researchers should give close attention to the physical effects of using the VIIRS/DNB NTL data for EPC estimation and other socio-economic studies. Besides, NTL is just one of components of EPC, other factors such as GDP, population, and urbanization also impact on EPC^46^. However, coarse statistical GDP and population data are unfeasible for fine scale estimation. Furthermore, using estimated gridded GDP and population product would introduce inevitable errors. Thus, GDP, population, and urbanization were not considered when EPC estimation in the present study.

## Conclusions

Artificial light is closely related to the EPC, therefore the NTL is a superior indicator of the EPC and is widely used for gridded EPC estimation^9,10^. However, the current VIIRS/DNB monthly NTL data did not meet high estimation accuracy requirements due to the impact of missing pixels and ephemeral light^57^. Thus, in this study, a more suitable interpolated NTL dataset was first proposed based on the cubic Hermite method, then the data were subjected to regression analysis. Moreover, few studies took the spatial non-stationary relationship between EPC and NTL into account in the estimation. In contrast to previous research^12,58^, this study has developed a classification regression method that based on the relationship between EPC and NTL.

Generally, the proposed approach resulted in a significant improvement in accuracy over OLS and GWR. The R^2^ values for the proposed method were greater than the OLS (0.900, 0.994 and 0.934 *vs*. 0.805). Furthermore, with regard to the RE values, the proposed method had more cities (145 vs. 72 and 86) that fell into the [− 25%, 25%] bin than OLS and GWR. In addition, by comparing estimation results with previous literature, results for this study showed a higher accuracy in many provinces. In conclusion, the comparative results for this study indicate that the approach used gives new insights into the explicit spatial distribution estimation of EPC based on the NTL. In addition to gridded EPC estimation, the proposed method also has a great potential for other socio-economic indicators estimation (e.g., GDP and carbon dioxide emission).
